# Prospective study of continuous rhythm monitoring in patients with early post-infarction systolic dysfunction: clinical impact of arrhythmias detected by an implantable cardiac monitoring device with real-time transmission—the TeVeO study protocol

**DOI:** 10.1136/bmjopen-2024-094764

**Published:** 2025-05-02

**Authors:** Jesus Hernandez-Hernandez, Alba Cruz-Galban, Olga Duran-Bobin, Javier Garcia-Seara, Teba Gonzalez-Ferrero, Jose Morinigo, Carlos González-Juanatey, Manuel Sanchez-Garcia, Gonzalo Fernandez-Palacios, Jose Seijas-Amigo, Juliana Elices, Javier Portales-Fernandez, Francisco Martin-Herrero, Ana García-Campos, Jose A Perez-Rivera, Ana Martin-Garcia, Marta Alonso-Fernandez-Gatta, Alfonso Macías, Paloma Perez-Espejo, Javier Garcia-Fernandez, Pedro L Sanchez, Javier Jimenez-Candil

**Affiliations:** 1 Hospital Universitario de Salamanca-IBSAL, Salamanca, Spain; 2 Cardiology, IBSAL-Hospital Universitario de Salamanca, Salamanca, Spain; 3 Cardiology, Hospital Universitario de Lugo, Lugo, Spain; 4 Hospital General Universitario Nuestra Señora del Prado, Talavera de la Reina, Spain; 5 Hospital Clínico Universitario de Santiago de Compostela Servicio de Cardiología, Santiago De Compostela, Spain; 6 Instituto de Investigación Biomédica de Santiago de Compostela, Spain (IDIS-SERGAS), Santiago de Compostela, Spain; 7 Medicine, Universidad de Salamanca, Salamanca, Spain; 8 Hospital Universitario de Burgos, Burgos, Castilla y León, Spain; 9 Hospital Universitario de Caceres, Caceres, Spain; 10 Cardiology, Hospital Universitario de Salamanca-IBSAL, Salamanca, Spain; 11 CIBERCV, Madrid, Comunidad de Madrid, Spain; 12 Cardiology, Hospital Universitario de Salamanca, Salamanca, CL, Spain

**Keywords:** Pacing & electrophysiology, Ischaemic heart disease, Defibrillators

## Abstract

**ABSTRACT:**

**Introduction:**

Updated primary prevention strategies are needed for post-infarction sudden cardiac death (SCD) based on implantable cardioverter-defibrillator (ICD). Current recommendations, based on left ventricular systolic function and functional class, may be obsolete because they are derived from ancient studies that do not incorporate the potential benefit of either current comprehensive treatment of ischaemic heart disease or modern device programming. Among patients with post-infarction left ventricular dysfunction, modern implantable cardiac monitoring devices (ICM) allow a unique opportunity to determine in real-time the burden of non-sustained ventricular tachycardias and their relationship to the subsequent occurrence of sustained or symptomatic events.

**Methods and analysis:**

Approximately 200 patients with left ventricular ejection fraction (LVEF) equal to or less than 40% after acute myocardial infarction will be included in the study. They will be implanted with a Confirm RX, an ICM with real-time remote connection via a smartphone. At 6 months, LVEF and functional status will be re-evaluated and cardiac morpho-functional characterisation will be performed by MRI. At this time, and following current European guidelines, patients with an indication will receive an ICD; the others will continue to be monitored using an ICM for a minimum of 2 years. Patients are expected to be followed up for 4 years after the index event. More than 20 000 remote transmissions are expected to be analysed. The study will focus on the relationship between the detection of non-sustained ventricular tachycardias by ICMs (defined as at least 8 R-R intervals at 160 beats per minute) and the subsequent occurrence of symptomatic arrhythmic events. An advanced statistical analysis will be performed using machine and deep learning techniques to determine the clinical variables, those that are derived from monitoring and imaging tests and related to mid-term prognosis.

**Ethics and dissemination:**

The study was approved by the Ethical Committee of the University Hospital of Salamanca (protocol number PI 2019 03 246) on 30 April 2020. Each patient will be informed about the study in both oral and written form by a physician and will be included in the study after written consent is obtained.

For the first time, a study will provide real-time information on the arrhythmic burden of patients with post-infarction ventricular dysfunction and its prognostic implications in the medium term. Several publications in scientific journals are planned.

**Trial registration number:**

NCT04765943.

**STRENGTHS AND LIMITATIONS OF THIS STUDY**

- To evaluate the feasibility of detecting arrhythmias in post-infarction patients using an implantable cardiac monitoring device (ICM) with real-time transmission via Bluetooth.
- To determine in these patients the true incidence, burden and timing of cardiac arrhythmias and related symptoms using a smartphone.
- To analyse the prognostic impact of the burden of a non-sustained and asymptomatic ventricular tachycardia and its relationship with the evolution of the arrhythmic substrate determined by cardiac MRI.
- The use of costly and invasive ICMs may need to be studied in larger post-infarction populations to allow for subgroup analyses and to generalise our findings.

## Introduction

 Cardiovascular diseases are the leading cause of mortality in economically developed countries, being responsible for nearly one out of three demises.^1 2^ They include pathologies producing a huge impact on the quality of life of patients and their families and a major sociosanitary burden for national health systems. Among these diseases, ischaemic heart diseases, specifically acute myocardial infarction (AMI), are the leading cause of death.^3^

Among patients with AMI treated with primary angioplasty, the majority of sustained ventricular tachyarrhythmias (VTs) occur within 24 hours of presentation with symptoms.^4^ Once the acute phase of AMI has passed, the resulting scar may produce both alterations in segmental contraction leading to heart failure and a substrate for the development of VT. VTs cause SCD in up to 30% of AMI survivors.^5^ Interventions carried out to avoid SCD following VT are based on restoring sinus rhythm as soon as possible. Therefore, preventive strategies based on implantable cardioverter-defibrillators (ICDs) are the best clinical option for some selected patients. Several multicentric prospective clinical studies^6^,^8^ have shown that ICD implantation improves the overall survival of patients at high risk of developing a first episode of VT after AMI, a strategy known as primary prevention. As a general conclusion, mortality is reduced by around 20–30% and mainly due to avoidance of SCD. Although different approaches have been considered to identify those patients at higher risk, current scientific evidence only supports the selection of ICD candidates for primary prevention according to the following criteria:^5^ (a) left ventricular ejection fraction (LVEF) and (b) functional class according to the New York Heart Association scale, both determined between 40 days and 3 months after the AMI and under optimal pharmacological treatment.

Alternatively, non-sustained ventricular tachycardias (NSVT), which are usually asymptomatic, and especially when repeated over time, often precede sustained VT.^9^ These NSVT are the clinical manifestation of an underlying arrhythmogenic substrate and its activation by triggering factors. In fact, in different studies on patients with an ICD, the occurrence of NSVT is associated with a higher incidence of appropriate therapies.^9 10^

## Methods and analysis

### Study description

The TeVeO study is an observational, multicentre and prospective study involving six tertiary hospitals from Spain: Hospital Universitario de Salamanca, Hospital Universitario de Lugo, Hospital Universitario de Santiago, Hospital Universitario de Burgos, Hospital Universitario de Cáceres and Hospital de Talavera de la Reina. According to official data for 2020, the reference population of these institutions is approximately 2.5 million people.

The TeVeO project aims to study arrhythmias that occur early after AMI to predict the medium to long-term risk of developing potentially lethal VTs. The project is grounded on the main assumption that NSVT that spontaneously occur during the first 6 months after an AMI can predict short- to long-term functional outcomes, specifically the risk of the occurrence of sustained and symptomatic VT. Therefore, the analysis of these arrhythmic events, detected using an implantable cardiac monitoring device (ICM), could contribute to patient stratification and help to develop further improvements for patient management.

The TeVeO study assumes the following further hypotheses for studying NSVT following an AMI:

- ICMs can accurately detect NSVTs.
- There are differences in the arrhythmogenic substrate of patients presenting NSVT in the first 6 months after an AMI with depressed LVEF that can be determined by studying the scar tissue using cardiac MRI.

### Study population

All consecutive patients after an AMI with a LVEF ≤ 40% determined from the fourth day after the onset of the episode will be included.

#### Inclusion criteria

- AMI patients over 18 years old, regardless of their initial clinical presentation.
- LVEF ≤40% determined by transthoracic echocardiography 4 days after the onset of the AMI.
- Revascularisation during hospitalisation according to the clinical practice guidelines.
- Signed informed consent (see online supplemental material).

#### Exclusion criteria

- Non-ischaemic aetiology of left ventricular dysfunction.
- Patient already implanted with a cardiac device (pacemaker, ICD or cardiac resynchronisation device).
- Indication of pacemaker, ICD or cardiac resynchronisation device implantation during hospitalisation.
- Allergy or hypersensitivity to any ICM component.
- Contraindication for cardiac MRI performance.
- Life expectancy under 1 year due to a non-cardiac cause.
- Concomitant valvulopathy with an indication for surgery.
- Functional Class IV (NYHA) at discharge.
- No possibility to connect to remote monitoring.

#### Sample size calculation

The study aims to include around 200 AMI patients. Sample size has been estimated for a confidence level of 95%, defined by a coefficient Z_a_=1.96 and considering a prevalence (*p*) of NSVT (< 5 episodes in 6 months) of 15% and a precision level (*d*) of 5%. According to the following formula, in which *q=1* p:


$$n=Z_{a}^{2}\times p\times q/d^{2}$$

n=3.84 * 0.15 * 0.85/0.0025 = 196 patients to be included.

### Study design

Epidemiological and basic clinical data of all the patients hospitalised in the participating hospitals after an episode of AMI will be collected. Patients accomplishing inclusion and exclusion criteria will be submitted to continuous electrocardiographic monitoring until medical discharge. Before discharge, an ICM will be implanted. Optionally, and before ICM implantation, a cardiac MRI will be performed on days 6–10 after an AMI.

Patients will be reevaluated 6 months after the coronary revascularisation performed during hospitalisation. This clinical evaluation will include, at least, the clinical and functional status (NYHA scale), pharmacological treatment and LVEF determination by echocardiography and cardiac MRI. Table 1 summarises the clinical variables to be evaluated. Tables2  3 describe the data recorded from echocardiograms and cardiac MRIs, respectively. If the patient fulfils the current criteria of the European Society of Cardiology according to LVEF criteria and functional class,^5^ an ICD will then be implanted. If the patient does not fulfil the ICD criteria, ICM monitoring will continue for an additional 24 months. According to scientific evidence, 5–15% of subjects with LVEF <40% in the first week post-AMI with revascularisation will recover part of the ventricular function, and thus fall outside of the criteria for receiving an ICD.

**Table 1:** Clinical data to be collected as part of the TeVeO study

Variable	At hospital discharge	6 months	24 months
Demographics	√	√	√
Risk factors	√	√	√
ECG*	√	√	√
Location of AMI	√		
Coronary anatomy	√		
Revascularisation	√		
Medical treatment	√	√	√
Functional class (NYHA)	√	√	√

* Atrial rhythm (sinus, atrial fibrillation, atrial flutter), QRS duration, number of Q waves, presence and type of bundle branch block.

AMI, acute myocardial infarction; NYHA, New York Heart Association.

**Table 2:** Data from echocardiograms to be collected as part of the TeVeO study

Variable	≥ 4 days after AMI	6 months
Left ventricular (LV) end-diastolic volume index, mL/m^2^	√	√
Left ventricular (LV) end-systolic volume index, mL/m^2^	√	√
Left ventricular ejection fraction, %	√	√
Mitral regurgitation*	√	√

* Degree of mitral regurgitation: none, mild, moderate, severe.

AMI, acute myocardial infarction.

**Table 3:** Data from cardiac MRI to be collected as part of the TeVeO study

Variable	During hospitalisation	At six months
Left ventricular end-diastolic volume index, mL/m^2^	√	√
Left ventricular end-systolic volume index, mL/m^2^	√	√
Left ventricular ejection fraction, %	√	√
Late gadolinium enhancement mass (2 SD), g	√	√
Late gadolinium enhancement mass (5 SD), g	√	√
Transmural late gadolinium enhancement	√	√
Late gadolinium enhancement distribution	√	√
Late gadolinium enhancement location*	√	√
Intraventricular thrombus	√	√
T1 mapping, ms†	√	
T2 mapping, ms†	√	
Extracellular volume fraction†, %	√	
Microvascular obstruction	√	

* Anterior, inferior, septal, lateral.

† Determined in the core zone of the infarct, in the peri-infarct zone and in remote zones

**Figure 1:**
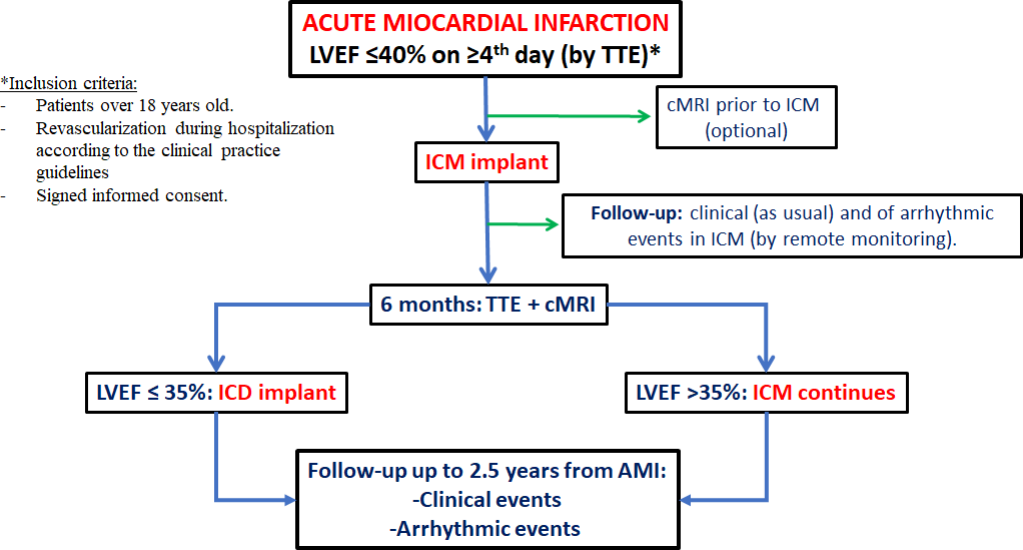
Descriptive graph of patient flow. AMI, acute myocardial infarction; cMRI, cardiac MRI; ICD, implantable cardioverter-defibrillator; ICM, implantable cardiac monitoring device; LVEF, left ventricular ejection fraction; TTE, transthoracic echocardiogram.

Patients will be followed up during the first 2.5 years after the AMI. Periodic clinical evaluation will be performed according to the protocols of every centre. Both devices (ICM and ICD) will be followed by remote monitoring. Figure 1 shows the flow of patients in the study. Patient management will be performed according to current European Guidelines. Patient enrolment in the study began in August 2020 and completion of the follow-up procedure is scheduled for 30 June 2025. An online database will be created for the incorporation of data related to the study, in which the patients will be anonymised.

### Objectives

The main objective of the TeVeO study is to assess the burden of NSVT occurring in the first 6 months after AMI and to determine its association with the subsequent occurrence of sustained VT or sudden cardiac death.

In addition, secondary objectives have been established:

- To study the feasibility and accuracy of an ICM to register NSVT following an AMI in patients with a relevant systolic dysfunction.
- To analyse if there are differences in the substrate in patients with/without NSVT following an AMI (scar, compact necrosis and heterogeneous tissue extensions) by cardiac MRI and gadolinium delayed enhancement.
- To evaluate the long-term prognostic impact (2.5 years) of the episodes of NSVT produced during the first 6 months after an AMI: cardiovascular mortality, hospitalisations due to cardiac cause (heart failure, acute coronary syndrome and arrhythmia).

### ICM implantation technique, programming and remote monitoring

The Confirm RX DM3500 (Abbott, USA) will be used as the ICM system. The ICM will be inserted under the skin in the left pectoral region. Recommended insertion locations are as follows:

- 4th intercostal space, 45° relative to the sternum, along the axis of the heart.
- 4th intercostal space, parallel to the sternum.
- anterolateral, inframammary between the fifth and sixth ribs.

**Figure 2:**
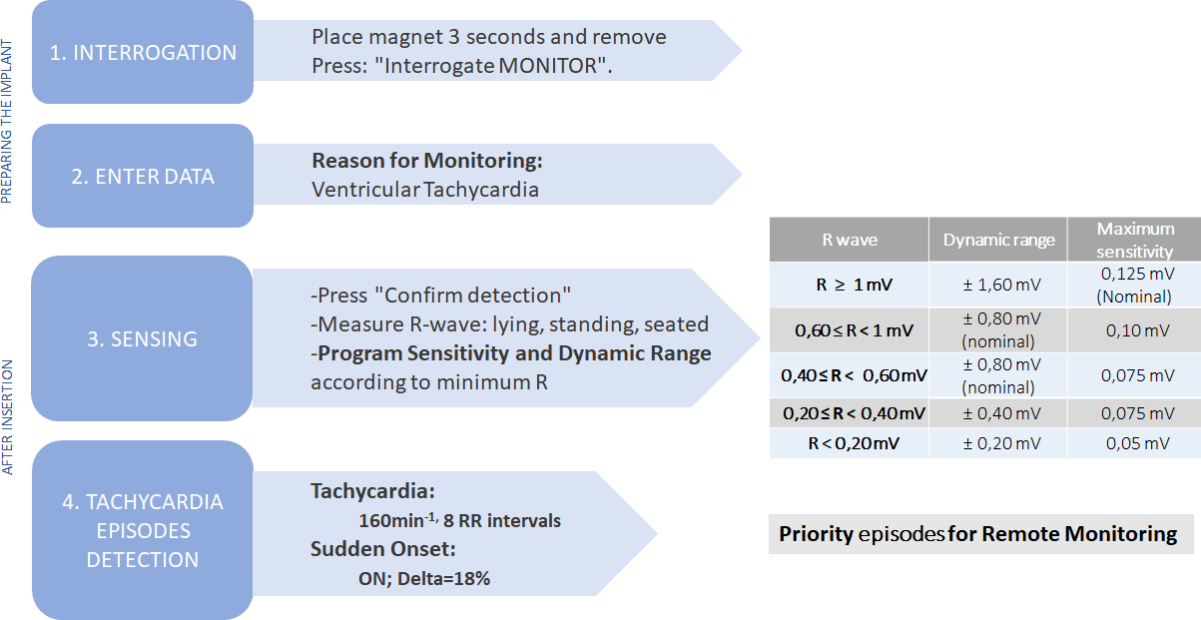
Programming of the Confirm RX device according to the TeVeO study.

The programming of the ICMs is summarised in figure 2. Briefly, the devices will be programmed to detect ventricular tachycardias of at least eight R-R intervals (which is the shortest duration of an episode allowed by the device) at a rate equal to or greater than 160 beats per minute.^9^ Through the myMerlin mobile application, and using Bluetooth technology, the device transmits all symptomatic or programming-defined arrhythmic events. These events will be available for online analysis at MyMerlin.net. Patient-triggered symptom transmissions and episodes of tachycardia will be scheduled as a priority. In addition, a monthly remote transmission will be scheduled by default.

### ICD programming

Detection and therapy programming will be standardised and will include two zones^11^: Ventricular fibrillation (cycle length (CL) < 250 ms) and ventricular tachycardia zone 1 (CL from 250 to 320 ms). In both cases, tachycardia detection will require that 30 of the last 40 R-R intervals have a CL below the cut-off point. Episodes classified as ventricular fibrillation will receive a sequence of high-energy shocks. Otherwise, episodes detected in ventricular tachycardia zone 1 will be initially treated by a single antitachycardia pacing sequence (an 8-pulse-burst train at 88% or 5-pulse-burst train at 84%^12^). Failed antitachycardia pacing therapy will be followed by shock and then other shocks, as necessary. An additional monitoring zone (ventricular tachycardia zone 2) will be programmed for episodes with a CL from 321 to 400 ms.

All devices will be programmed to store the far-field electrograms before the onset of the episodes detected to aid in rhythm classification.

### MRI protocol

All patients will undergo contrast-enhanced cardiac MRI using a 1.5 T or 3 T scanner. All images will be obtained with electrocardiographic gating and breath-holding. The protocol will include standard segmented cine steady-state free-precession, T2W-STIR, T2 mapping, native and post-contrast T1 mapping and late gadolinium enhancement (LGE) sequences. LGE images will be obtained 10–15 min after injection of 0.15 mmol gadobutrol contrast agent per kg body weight (Gadovist, Bayer HealthCare Pharmaceuticals, USA). LGE images will be used for infarct characterisation. Infarct and heterogeneous tissue (ie, grey zone) mass will be quantified by two methods: (1) the full width at half maximum and (2) based on the SD of the signal intensity from the remote mean healthy myocardium (> 2 SDs defines the total infarct mass, >5 SDs the core infarct and heterogeneous tissue between 2 and 5 SDs).

### Definitions

- NSVT: any rhythm with a wide QRS from a ventricular origin that is self-terminated within 30 s, with a heart rate over 100 beats per minute and over five beats.^5^
- Sustained VT: any rhythm with a wide QRS from a ventricular origin with a heart rate over 100 beats per minute, which lasts over 30 s or that needs an intervention to be finished.^5^
- Monomorphic VT: all QRS complexes present the same morphology.^5^
- Polymorphic VT: QRS morphology changes in any heartbeat.^5^
- Appropriate therapy: any therapy (antitachycardia pacing or shock) applied by ICD due to a ventricular tachyarrhythmia.^5^
- Inappropriate therapy: any ICD therapy (antitachycardia pacing or shock) that is not due to a ventricular tachyarrhythmia.^5^
- Ischaemic left ventricular dysfunction: systolic dysfunction due to previous coronary artery disease with myocardial necrosis, with previous signs of at least one obstructed epicardial coronary artery and the presence of an ischaemic scar in the cardiac MRI with characteristic distribution of LGE.

### Patient and public involvement

Patients or the public were not involved in the design, conduct, reporting or dissemination plans of our research.

## Ethics and dissemination

### Quality control

Different processes will be carried out to guarantee study data quality and thus maximise the validity and reliability of the measurements of the results (see SPIRIT Check List, online supplemental material). To this effect, fieldwork operation manuals have been prepared. These documents specify the adequate procedure for performing each test. All of these actions will confirm the adequate performance of each procedure. Monthly meetings will be held with the principal investigator of the study to analyse the entire process, a monthly newsletter will be sent and an annual report on the progress of the study will be prepared.

The analysis of the arrhythmic and clinical events will be performed by a committee composed of two investigators (JH-H and JP-F). In case of discrepancy in diagnosis, the analysis of a third investigator (JM) will be requested.

### Ethical review board and dissemination plan

The study was approved by the Ethical Committee of the University Hospital of Salamanca (protocol number PI 2019 03 246) on 30 April 2020. Participants will be required to sign an informed consent form prior to participation in the study, in accordance with the Declaration of Helsinki and WHO standards for observational studies. These actions will be the responsibility of the principal investigator of each centre. Participants will be informed of the objectives of the project and the risks and benefits of the examinations made. None of the examinations poses life-threatening risks for the type of participants to be included in the study.

The study protocol, including variables and prespecified research questions, was registered at Clinical Trial Registration NCT04765943 and approved on 18 February 2021. This manuscript reflects the final version of the protocol. After the follow-up period is completed, the database will be closed, statistical work will be carried out, and the results will be interpreted and disseminated in a scientific journal.

### Data statement

Our data will be accessible at the Institute of Research of the University Hospital of Salamanca. Furthermore, our dataset will be published in a public repository. An additional code for our spatial analysis will be shared in a public GitHub repository.

## Discussion

This is the first study in which patients with post-infarction ventricular dysfunction will be monitored for a prolonged period employing an implantable device with Bluetooth connection and remote monitoring capability. The Confirm RX device, which connects to a smartphone application, enables real-time recording of arrhythmias and symptoms, facilitating remote analysis and empowering patients in their clinical process. It thus represents a clinical and technological update to previous studies, such as the CARISMA study,^13^ in which long-term monitoring by implantable devices did not allow real-time analysis by remote control.

The main objective of the study, to analyse the NSVT burden during the first months after infarction, will provide information on the evolution of the arrhythmic substrate after an acute ischaemic event, to improve risk stratification for sudden death and sustained ventricular arrhythmias. Sustained ventricular arrhythmias present a characteristic chronology after ST-elevation acute myocardial infarction. The current incidence of post-infarction monomorphic VT is estimated to be 1–3%.^14^ Two peaks are observed in the incidence of monomorphic VT after ST-elevation myocardial infarction: a first peak in the first 3 months, associated with the coexistence of severe heart failure and an estimated 2-year survival of 40–50%, and a second peak after 3 years, with a more benign course.^15^ In this regard, several studies conducted between the end of the 1990s and early 2000s have shown that patients with ventricular dysfunction objectified at least 40 days to 3 months after AMI benefit from ICD implantation because it increases survival by reducing sudden death.

Since then, the management of heart failure patients has changed dramatically with the advent of a series of novel drug classes that reduce not only mortality but specifically SCD rates.^16^ In addition, contemporary studies report a dramatic reduction in the rates of therapies delivered by prophylactic defibrillators from initially 17% per year in MADIT II to currently 1–3% per year.^17^ At the same time, the complication rates associated with the defibrillator therapy remain significant without obvious decrease.^18^ Thus, there is a significant amount of evidence showing that the risk-benefit of defibrillator implantation for primary prevention of SCD in patients with severely reduced LVEF has substantially changed since these two landmark trials were conducted 20–25 years ago.

Therefore, it seems necessary to incorporate new tools in the risk stratification of ventricular arrhythmias after a myocardial infarction. Accordingly, imaging techniques with tissue characterisation by cardiac MRI are promising because scar characteristics analysed by late gadolinium enhancement have proved to be a strong predictor of appropriate therapies.^19^ The relationship between the incidence and burden of post-infarction NSVT and the subsequent risk of ventricular arrhythmias needs to be explored. Indeed, patients with high NSVT burden are at high risk of appropriate therapies for what appears to be the same ventricular arrhythmia increasing in duration over time,^20^ which could be an expression of a progressive evolution process of the arrhythmic substrate.^21^

Finally, in the CARISMA study, almost half of post-AMI patients had clinically relevant arrhythmias.^13^ Since then, the pharmacological and invasive management of these patients has undergone significant improvements. The TeVeO study provides a unique opportunity to assess in a similar population whether these therapeutic modifications translate into a different arrhythmogenic expression.

## Supplementary material

10.1136/bmjopen-2024-094764 online supplemental file 2

## Data Availability

Data are available upon reasonable request.
