# Supplementary material for: Prospective study of continuous rhythm monitoring in patients with early post-infarction systolic dysfunction: clinical impact of arrhythmias detected by an implantable cardiac monitoring device with real-time transmission—the TeVeO study protocol
Source: BMJ Open. 2025 May 2;15(5):e094764. doi: 10.1136/bmjopen-2024-094764 (PMC12049914; doi:10.1136/bmjopen-2024-094764)
Supplement: online supplemental file 2 [file bmjopen-15-5-s001.docx]

**SPIRIT Check List**

**Administrative information**

Title

1. Descriptive title identifying the study design, population, interventions, and, if applicable, trial acronym. **Page 1**.

Trial registration

2a Trial identifier and registry name. **Pages 3, 14.**

2b All items from the World Health Organization Trial Registration Data Set. **Page 1 to 14.**

Protocol version

3 Date and version identifier. **Page 14.**

Funding:

4 Sources and types of financial, material, and other support. **Page 22.**

Roles and responsibility.

5 a. Names, affiliations, and roles of protocol contributors. **Pages 21-22**.

5c. Name and contact information for the trial sponsor. **Not applicable.**

5c. Role of study sponsor and funders, if any, in study design; collection, management, analysis, and interpretation of data; writing of the report; and the decision to submit the report for publication, including whether they will have ultimate authority over any of these activities. **Page 23**.

5d Composition, roles, and responsibilities of the coordinating centre, steering committee, endpoint adjudication committee, data management team, and other individuals or groups overseeing the trial, if applicable (see Item 21a for data monitoring committee). **Page 13.**

**Introduction**

Background and rationale

6a. Description of research question and justification for undertaking the trial, including summary of relevant studies (published and unpublished) examining benefits and harms for each intervention. **Pages 6,7.**

6b Explanation for choice of comparators. **Not applicable.**

Objectives

7 Specific objectives or hypotheses. **Page 7 and page 10**.

Trial design

8 Description of trial design including type of trial (eg, parallel group, crossover, factorial, single group), allocation ratio, and framework (eg, superiority, equivalence, noninferiority, exploratory). **Page 7.**

**Methods: Participants, interventions, and outcomes**

Study setting

9 Description of study settings (eg, community clinic, academic hospital) and list of countries where data will be collected. Reference to where list of study sites can be obtained. **Page 1 and page 7**.

Eligibility criteria

10 Inclusion and exclusion criteria for participants. If applicable, eligibility criteria for study centres and individuals who will perform the interventions (eg, surgeons, psychotherapists). **Page 8.**

Interventions

11a Interventions for each group with sufficient detail to allow replication, including how and when they will be administered. **Page 9.**

11b Criteria for discontinuing or modifying allocated interventions for a given trial participant (eg, drug dose change in response to harms, participant request, or improving/worsening disease). **Not applicable**.

11c Strategies to improve adherence to intervention protocols, and any procedures for monitoring adherence (eg, drug tablet return, laboratory tests). **Not applicable.**

11d Relevant concomitant care and interventions that are permitted or prohibited during the trial. **Page 10.**

Outcomes

12 Primary, secondary, and other outcomes, including the specific measurement variable (eg, systolic blood pressure), analysis metric (eg, change from baseline, final value, time to event), method of aggregation (eg, median, proportion), and time point for each outcome. Explanation of the clinical relevance of chosen efficacy and harm outcomes is strongly recommended. **Page 10.**

Participant timeline

13 Time schedule of enrolment, interventions (including any run-ins and washouts), assessments, and visits for participants. A schematic diagram is highly recommended (see Figure). **Page 9 and figure 1.**

Sample size

14 Estimated number of participants needed to achieve study objectives and how it was determined, including clinical and statistical assumptions supporting any sample size calculations. **Page 8 and 9**.

Recruitment

15 Strategies for achieving adequate participant enrolment to reach target sample size. **Not applicable.**

**Methods: Assignment of interventions (for controlled trials)**

Allocation: Sequence generation

16a Method of generating the allocation sequence (eg, computer-generated random numbers), and list of any factors for stratification. To reduce predictability of a random sequence, details of any planned restriction (eg, blocking) should be provided in a separate document that is unavailable to those who enrol participants or assign interventions. **Not applicable.**

Allocation concealment mechanism

16b Mechanism of implementing the allocation sequence (eg, central telephone; sequentially numbered, opaque, sealed envelopes), describing any steps to conceal the sequence until interventions are assigned. **Not applicable.**

Implementation

16c Who will generate the allocation sequence, who will enrol participants, and who will assign participants to interventions. **Not applicable.**

Blinding (masking)

17a Who will be blinded after assignment to interventions (eg, trial participants, care providers, outcome assessors, data analysts), and how. **Not applicable.**

17b If blinded, circumstances under which unblinding is permissible, and procedure for revealing a participant’s allocated intervention during the trial. **Not applicable.**

**Methods: Data collection, management, and analysis**

Data collection methods

18a Plans for assessment and collection of outcome, baseline, and other trial data, including any related processes to promote data quality (eg, duplicate measurements, training of assessors) and a description of study instruments (eg, questionnaires, laboratory tests) along with their reliability and validity, if known. Reference to where data collection forms can be found, if not in the protocol. **Page 10 and page 13.**

18b Plans to promote participant retention and complete follow-up, including list of any outcome data to be collected for participants who discontinue or deviate from intervention protocols. **Not applicable.**

Data management

19 Plans for data entry, coding, security, and storage, including any related processes to promote data quality (eg, double data entry; range checks for data values). Reference to where details of data management procedures can be found, if not in the protocol. **Page 10 and page 13.**

Statistical methods

20a Statistical methods for analysing primary and secondary outcomes. Reference to where other details of the statistical analysis plan can be found, if not in the protocol. **Page 3**

20b Methods for any additional analyses (eg, subgroup and adjusted analyses). **Page 3.**

20c Definition of analysis population relating to protocol non-adherence (eg, as randomised analysis), and any statistical methods to handle missing data (eg, multiple imputation). **Not applicable.**

**Methods: Monitoring**

Data monitoring

21a Composition of data monitoring committee (DMC); summary of its role and reporting structure; statement of whether it is independent from the sponsor and competing interests; and reference to where further details about its charter can be found, if not in the protocol. Alternatively, an explanation of why a DMC is not needed. **Page 13.**

21b Description of any interim analyses and stopping guidelines, including who will have access to these interim results and make the final decision to terminate the trial. **Not applicable.**

Harms

22 Plans for collecting, assessing, reporting, and managing solicited and spontaneously reported adverse events and other unintended effects of trial interventions or trial conduct. **Page 13.**

Auditing

23 Frequency and procedures for auditing trial conduct, if any, and whether the process will be independent from investigators and the sponsor. **Page 13.**

**Ethics and dissemination**

Research ethics approval

24 Plans for seeking research ethics committee/institutional review board (REC/IRB) approval. **Pages 13-14.**

Protocol amendments

25 Plans for communicating important protocol modifications (eg, changes to eligibility criteria, outcomes, analyses) to relevant parties (eg, investigators, REC/IRBs, trial participants, trial registries, journals, regulators). **Page 14.**

Consent or assent

26a Who will obtain informed consent or assent from potential trial participants or authorised surrogates, and how (see Item 32). **Page 14.**

26b Additional consent provisions for collection and use of participant data and biological specimens in ancillary studies, if applicable. **Not applicable.**

Confidentiality

27 How personal information about potential and enrolled participants will be collected, shared, and maintained in order to protect confidentiality before, during, and after the trial. **Pages 10, 13 and 14.**

Declaration of interests

28 Financial and other competing interests for principal investigators for the overall trial and each study site. **Page 22.**

Access to data

29 Statement of who will have access to the final trial dataset, and disclosure of contractual agreements that limit such access for investigators. **Page 14**.

Ancillary and post-trial care

30 Provisions, if any, for ancillary and post-trial care, and for compensation to those who suffer harm from trial participation. **Not applicable.**

Dissemination policy

31a Plans for investigators and sponsor to communicate trial results to participants, healthcare professionals, the public, and other relevant groups (eg, via publication, reporting in results databases, or other data sharing arrangements), including any publication restrictions. **Page 14.**

31b Authorship eligibility guidelines and any intended use of professional Writers. **Page 22.**

31c Plans, if any, for granting public access to the full protocol, participant-level dataset, and statistical code. **Page 14.**

**Appendices**

Informed consent materials

32 Model consent form and other related documentation given to participants and authorised surrogates**. Pages 13-14.** **Supplementary material.**

Biological specimens

33 Plans for collection, laboratory evaluation, and storage of biological specimens for genetic or molecular analysis in the current trial and for future use in ancillary studies, if applicable. **Not applicable.**
