# Supplementary material for: Prospective study of continuous rhythm monitoring in patients with early post-infarction systolic dysfunction: clinical impact of arrhythmias detected by an implantable cardiac monitoring device with real-time transmission—the TeVeO study protocol
Source: BMJ Open. 2025 May 2;15(5):e094764. doi: 10.1136/bmjopen-2024-094764 (PMC12049914; doi:10.1136/bmjopen-2024-094764)
Supplement: Supplementary file 2 [file bmjopen-2024-094764.reviewer_comments.pdf]

PEER REVIEW HISTORY

BMJ Open publishes all reviews undertaken for accepted manuscripts. Reviewers are asked to complete a checklist review form and are provided with free text boxes to elaborate on their assessment. These free text comments are reproduced below.

ARTICLE DETAILS

Title (Provisional)

A prospective study of continuous rhythm monitoring in patients with early post-infarction systolic dysfunction: clinical impact of arrhythmias detected by an implantable cardiac monitoring device with real-time transmission. The TeVeO study protocol.

Authors

Hernandez-Hernandez, Jesus; Cruz-Galban, Alba; Duran-Bobin, Olga; Garcia-Seara, Javier; Gonzalez-Ferrero, Teba; Morinigo, Jose; González-Juanatey, Carlos; Sanchez-Garcia, Manuel; Fernandez-Palacios, Gonzalo; Seijas-Amigo, Jose; Elices, Juliana; Portales-Fernandez, Javier; Martin-Herrero, Francisco; García-Campos, Ana; Perez-Rivera, Jose A; Martin-Garcia, Ana; Alonso-Fernandez-Gata, Marta; Macías, Alfonso; Perez-Espejo, Paloma; Garcia-Fernandez, Javier; Sanchez, Pedro L; Jimenez-Candil, Javier

---

VERSION 1 - REVIEW

**Reviewer**	**1**
**Name**	**Verma, Narsingh**
**Affiliation**	**King George Medical University, Physiology**
**Date**	**05-Jan-2025**
**COI**	**None**

This study is first of its kind and it was carried out in well defined manner

---

**Reviewer**	**2**
**Name**	**Pimentel, Mauricio**
**Affiliation**	**Hospital de Clinicas de Porto Alegre, Cardiology**
**Date**	**09-Mar-2025**
**COI**	**None**

General comment:

The authors present an elegant design for a Prospective Study of Continuous Rhythm Monitoring using an Implantable Cardiac Monitoring Device in Patients with Post-Infarction Systolic Dysfunction. The issue of risk stratification for ventricular arrhythmias after a myocardial infarction is clinically relevant and remains incompletely resolved.

Minor revisions needed:

The authors should explain why the definition of non-sustained ventricular tachycardia (NSVT) by ICMs was set at at least 8 R-R intervals at 160 bpm.

Clarify whether patients with cardiogenic shock at the initial clinical presentation will be included.

Justify why the timing for re-evaluation was set at 6 months rather than 3 months after AMI.

Does the study design establish an event adjudication committee to assess all ICM records, ICD records, and other clinically relevant events?

VERSION 1 - AUTHOR RESPONSE

Reviewer 1.

Dr. Narsingh Verma, King George Medical University

Comments to the Author:

This study is first of its kind and it was carried out in well defined manner

Thank you for your comment.

Reviewer: 2

Dr. Mauricio Pimentel, Hospital de Clinicas de Porto Alegre

Than you for your comments and suggestions.

Comments to the Author:

General comment:

The authors present an elegant design for a Prospective Study of Continuous Rhythm Monitoring using an Implantable Cardiac Monitoring Device in Patients with Post-Infarction Systolic Dysfunction. The issue of risk stratification for ventricular arrhythmias after a myocardial infarction is clinically relevant and remains incompletely resolved.

Minor revisions needed:

1. The authors should explain why the definition of non-sustained ventricular tachycardia (NSVT) by ICMs was set at at least 8 R-R intervals at 160 bpm.

The programming of NSTV detection was done in this way in ICMs for two reasons: 1- scientific consistency, given that in our experience from 150-160 bpm NSVT episodes are clinically relevant (Jimenez-Candil et al. Am J Cardiol 2016); 2- the shortest programmable duration of a tachycardia in these devices is 8 RR intervals. Please, see page 11, first paragraph.

2. Clarify whether patients with cardiogenic shock at the initial clinical presentation will be included.

All patients with AMI were included, regardless of their initial clinical presentation. Only subjects who remained in functional class IV at discharge were excluded. Please see page 8, inclusion and exclusion criteria of the revised manuscript.

3. Justify why the timing for re-evaluation was set at 6 months rather than 3 months after AMI.

The rationale for delaying LVEF reassessment is that it may facilitate LVEF recovery above the cut-off point at which guidelines recommend ICD implantation. Please, see page 9 of the rewieved manuscript.

4. Does the study design establish an event adjudication committee to assess all ICM records, ICD records, and other clinically relevant events?

Yes, we did it. Please page 13 of the rewieved manuscript.
